# Cutaneous metastasis to the face from colon adenocarcinoma. Case report

**DOI:** 10.1186/1477-7800-3-2

**Published:** 2006-02-02

**Authors:** Georgios Fyrmpas, Nikolaos Barbetakis, Andreas Efstathiou, Iordanis Konstantinidis, Christodoulos Tsilikas

**Affiliations:** 1 Department of Otolaryngology Head & Neck Surgery, Aristotle University of Thessaloniki, AHEPA Hospital, S. Kiriakidi 1, 546 36 Thessaloniki, Greece; 2 Department of Thoracic Surgery, Theagenio Cancer Hospital, Al. Simeonidi 2, Thessaloniki, Greece; 3 Department of Otolaryngology, Kavala General District Hospital, Kavala, Greece

## Abstract

**Background:**

Facial skin metastases from colorectal cancer are extremely rare and appear several years after resection of the primary tumour. They are an important finding, often being the first sign of metastasis from a previously treated colon cancer.

**Case presentation:**

We describe a case of a 69 year old patient with cutaneous metastasis to the chin from a previously treated adenocarcinoma of the colon. The patient presented with dyspnoea, pleuritic pain and loss of weight. A chest x-ray revealed a right upper lobe mass of the lung which on subsequent surgical exploration proved to be metastatic from colorectal adenocarcinoma resected three years ago. During the postoperative course, a nodule was noted on the chin and excision biopsy revealed it was also a metastasis from the initial colorectal cancer. Palliative chemoradiotherapy was administered and the patient survived 8 months.

**Conclusion:**

High index of suspicion is necessary for the early detection of facial cutaneous metastases from colorectal cancer. The aim is to start treatment as soon as possible before widespread visceral metastases occur. Cutaneous metastases from colorectal cancer carry a better prognosis in comparison to those of other epithelial tumours.

## Introduction

Cutaneous metastases in the facial region occur in less than 0.5% of patients with metastatic cancer and they usually originate from malignant melanoma [1]. Colon adenocarcinoma metastasises to the facial skin very rarely and only 4 cases have been reported in the English language literature to date [1-3]. In this report we describe an unusual case of colorectal cancer metastasising to the chin and lung.

## Case presentation

**Figure 1:**
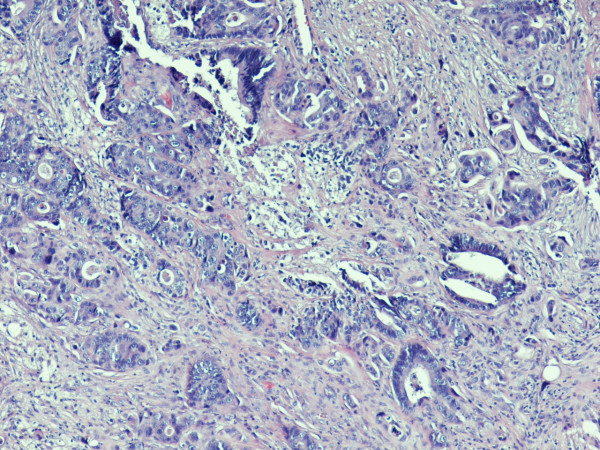
**Histological section from lung biopsy material**. Metastatic adenocarcinoma to the lung from previous colon cancer (haematoxylin-eosin ×200).

**Figure 2:**
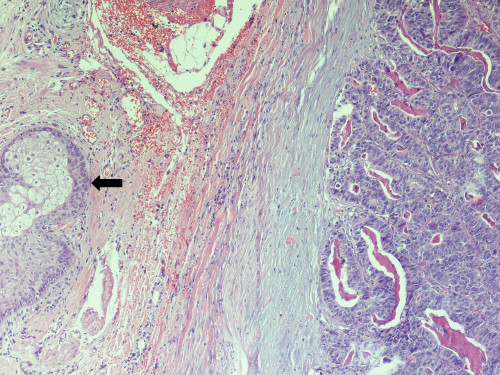
**Histological section from skin specimen**. Representative area of the skin lesion showing infiltration by moderately differentiated adenocarcinoma and a sebaceous gland (black arrow) (haematoxylin-eosin ×200).

A 69 year old patient presented with a short history of dyspnoea, pleuritic pain and loss of weight. He had undergone right hemicolectomy for adenocarcinoma of the large intestine 3 years ago. A right upper lobe mass was noted on the chest x-ray and carcinoembryonic antigen and CA 19-9 levels were raised. Exploratory thoracotomy revealed a mass infiltrating the superior vena cava and the carina which was therefore considered unresectable. Biopsies were taken and histology showed adenocarcinoma, apparently metastatic from the previous cancer of the colon [figure 1]. Further investigations for detecting metastases to other sites were negative. However, a whitish nodule measuring 6 mm was noted on the chin and the patient revealed that it had been present for three months. Excision biopsy of the facial lesion and subsequent histological sections showed skin infiltration by moderately differentiated adenocarcinoma [figure 2]. The patient was referred for chemoradiotherapy and survived 8 months.

## Discussion

Lung and breast cancers are the commonest epithelial malignancies metastasising to the skin in men and women respectively [4]. Clinically, cutaneous metastases manifest as nodules, ulceration, cellulitis like lesions, bullae or fibrotic processes. Histologically, they are classified as adenocarcinoma, squamous cell carcinoma, undifferentiated carcinoma and other miscellaneous types [5].

Colon adenocarcinoma most often metastasises to the liver and lung. Cutaneous metastases occur in less than 4% of all cases [6]. The most frequent site is the abdominal skin and specifically the area of previous surgical incisions [1]. Direct spread from the initial tumour or dissemination via lymphatics account for this type of metastasis. If veins are invaded, distant skin deposits may occur [7]. In the head and neck region only fifteen cases of cutaneous metastases have been reported [8].

Cutaneous metastases as a first sign of internal malignancy occur infrequently. More commonly, they are early indicators of metastatic disease [1]. Diagnosis may delay several months [9] unless the skin lesion grows rapidly or other sites such as the lung or liver are affected by tumour spread. In our case, the cutaneous metastasis went unnoticed for three months and it was the pulmonary symptoms that suggested recurrence of the previous colon adenocarcinoma. Early recognition of tumour relapse from a suspicious skin lesion may lead to initiation of treatment before widespread metastases occur [2].

In general, skin metastasis is a poor prognostic sign. If the primary tumour is the lung, the cervix or the oesophagus most patients die within three months. In the case of colorectal cancer, however, skin involvement is not a preterminal event [10]. Treatment involves radiotherapy or excision and patients may survive up to a year [3,10].

## Conclusion

Cutaneous metastases in the face from colorectal cancer are very rare and they may go unnoticed for a long period. Usually, they indicate tumour relapse several years after primary resection. Early detection requires high index of suspicion. Therefore, close inspection of new skin lesions in patients with a history of malignancy is imperative, and diagnostic biopsy is essential. Cutaneous metastasis is not a preterminal event and appropriate treatment may prolong patient survival up to a year.

## Competing interests

The author(s) declare that they have no competing interests.

## Authors' contributions

G. Fyrmpas, N. Barbetakis, A. Efstathiou, I. Konstantinidis took part in the care of the patient and contributed equally in carrying out the medical literature search and preparation of the manuscript. Ch. Tsilikas participated in the care of the patient and had the supervision of this report. All authors approved the final manuscript.

## References

[B1] Lookingbill DP, Spangler N, Helm KF (1993). Cutaneous metastases in patients with metastatic carcinoma: A retrospective study of 4020 patients. J Am Acad Dermatol.

[B2] Gottlieb JA, Schermer DR (1970). Cutaneous metastases from carcinoma of the colon. JAMA.

[B3] Stavrianos SD, McLean NR, Kelly CG, Fellows S (2000). Cutaneous metastasis to the head and neck from colonic carcinoma. Eur J Surg Oncol.

[B4] Kaplan RP (1986). Specific cutaneous manifestations of internal malignancy. Adv Dermatol.

[B5] Browstein MH, Helwig EG (1973). Spread of tumors to the skin. Arch Dermatol.

[B6] Abrams HL, Spiro R, Goldenstein N (1950). Metastases in carcinoma; analysis of 1000 autopsied cases. Cancer.

[B7] Browstein MH, Helwig EG (1972). Patterns of cutaneous metastasis. Arch Dermatol.

[B8] Lee M, Duke EE, Munoz J, Holaday L (1995). Colorectal cancer presenting with a cutaneous metastatic lesion on the scalp. Cutis.

[B9] Gmitter TL, Dhawan SS, Phillips MG, Wiszniak J (1990). Cutaneous metastases of colonic adenocarcinoma. Cutis.

[B10] Brady LW, O'Neill EA, Farber SH (1977). Unusual sites of metastases. Semin Oncol.

